# Low-temperature synthesis of colloidal few-layer WTe_2_ nanostructures for electrochemical hydrogen evolution

**DOI:** 10.1186/s11671-023-03796-7

**Published:** 2023-03-15

**Authors:** Rui Xie, Wenchen Luo, Luwei Zou, Xiulian Fan, Cheng Li, Tiezheng Lv, Jinming Jiang, Zhihui Chen, Yu Zhou

**Affiliations:** 1 School of Physics and Electronics, Hunan Key Laboratory of Nanophotonics and Devices, Central South University, 932 South Lushan Road, Changsha, 410083 Hunan People’s Republic of China; 2 College of Chemistry, Nankai University, Tianjin, 300071 People’s Republic of China; 3 Research Institute of Automobile Parts Technology, Hunan Institute of Technology, Hengyang, 421002 People’s Republic of China; 4 Powder Metallurgy Research Institute and State Key Laboratory of Powder Metallurgy, Central South University, Changsha, 410083 People’s Republic of China; 5 Department of Chemistry, Yale University, New Haven, CT 06511 USA; 6 Department of Basic Sciences, Air Force Engineering University, Xi’an, 710051 People’s Republic of China; 7 State Key Laboratory of Solidification Processing, Northwestern Polytechnical University, Carbon/Carbon Composites Research Center, Xi’an, 710072 People’s Republic of China; 8 State Key Laboratory of Structural Chemistry, Fujian Institute of Research on the Structure of Matter, Chinese Academy of Sciences, Fuzhou, 350002 Fujian People’s Republic of China

**Keywords:** Colloidal synthesis, Two-dimensional (2D) tellurides, Hydrogen evolution reaction, WTe_2_ nanostructures, Microelectrochemical reactor

## Abstract

High-quality transition metal tellurides, especially for WTe_2_, have been demonstrated to be necessarily synthesized under close environments and high temperatures, which are restricted by the low formation Gibbs free energy, thus limiting the electrochemical reaction mechanism and application studies. Here, we report a low-temperature colloidal synthesis of few-layer WTe_2_ nanostructures with lateral sizes around hundreds of nanometers, which could be tuned the aggregation state to obtain the nanoflowers or nanosheets by using different surfactant agents. The crystal phase and chemical composition of WTe_2_ nanostructures were analyzed by combining the characterization of X-ray diffraction and high-resolution transmission electron microscopy imaging and elements mapping. The as-synthesized WTe_2_ nanostructures and its hybrid catalysts were found to show an excellent HER performance with low overpotential and small Tafel slope. The carbon-based WTe_2_–GO and WTe_2_–CNT hybrid catalysts also have been synthesized by the similar strategy to study the electrochemical interface. The energy diagram and microreactor devices have been used to reveal the interface contribution to electrochemical performance, which shows the identical performance results with as-synthesized WTe_2_–carbon hybrid catalysts. These results summarize the interface design principle for semimetallic or metallic catalysts and also confirm the possible electrochemical applications of two-dimensional transition metal tellurides.

**Supplementary Information:**

The online version contains supplementary material available at 10.1186/s11671-023-03796-7.

## Introduction

Two-dimensional (2D) materials of layered metal chalcogenides have been widely investigated in the field of electronic, optoelectronic, ferroelectric and electrochemical devices applications [1–9]. Both theoretical and experimental studies of MoS_2_ catalysts have demonstrated transition metal dichalcogenides are amazing hydrogen evolution reaction (HER) model catalysts, which can be attributed to its low hydrogen adsorption Gibbs free energy at the edges and be low fabrication cost [10–12]. Thus, various strategies have been carried out to improve the density of active edge sites, design the interface and intralayer charge transfer process, or select a new type of high-catalytic-activity candidates [13–19]. The 2D metallic HER catalysts recently have been confirmed with high catalytic activity that is achieved through 2H-1 T′phase transition or selecting stable metallic phases, which benefit from the high electrical conductivities and intrinsic thermodynamic hydrogen adsorptions [18, 20–22]. Tungsten ditelluride (WTe_2_), as a new quantum matter, has been considered as a spin quantum Hall state, topological insulators and metallic ferroelectrics [23–25]. However, its electrochemical catalytic activities have not been systematically explored yet [26]. Currently, the limiting obstacle for its practical application is its large-scale synthesis. As discussed in our previous reports, low formation Gibbs free energy of WTe_2_ makes its low binding energy for the W–Te chemical bonds compared to other TMDs [27–29]. Several synthetic strategies have been shown for the synthesis of WTe_2_ bulk crystals and 2D forms, such as chemical vapor transport and chemical vapor deposition that requires high temperatures (> 800 °C) and close environments [27, 30]. However, low-temperature synthesis of WTe_2_ nanostructures with layers and morphology control is still absent. Theoretical calculations and experimental explorations have confirmed the stable phase of WTe_2_ and its potential electrochemical performance [26, 31, 32]. Meanwhile, the electrochemical interface design of metallic WTe_2_ nanoflakes is still unknown up to now. Hence, the absence of large-scale and low-temperature synthesis of WTe_2_ nanostructures and electrochemical interface experimental proof renders its wider energy application [33–36].

In this paper, we employed colloidal chemistry to control the morphology of WTe_2_ nanostructures at low-temperature synthesis conditions. It has been found that nanoflowers and nanosheets shaped WTe_2_ can be synthesized by tuning the surfactant agents. We also obtain the intrinsic HER activities of WTe_2_ nanostructures, presenting with similar performance with the MoS_2_ family materials. The importance of the interfacial charge transfer has also been explored by systematic study of carbon–WTe_2_ hybrid catalysts and by electrochemical microreactors. These results provide basis toward the low-temperature synthesis for the low-Gibbs-free-energy materials and a comprehensive understanding of design principles of the 2D TMDs HER catalytic activity.

## Experimental method

### Materials preparation

#### Preparation of WTe_2_ nanoflowers

0.1 mmol W(CO)_6_ was added into a 50-ml round-bottomed flask; then, 8 ml oleic acid and 2 ml oleyl amine were added into the same flask and mixed with W(CO)_6_. The air was pumped out and N_2_ gas flow was introduced for 3 times. Then the flask was heated to 120 °C and kept for 10 min with N_2_ continuously flowing. Then the temperature was raised to 240 °C and 2 ml TOP-Te (1 mol L^−1^) was injected into the flask at the rate of 1 ml/min. Then the flask was heated to 300 °C and kept for 30 min. After cooling down to room temperature, the mixed solution was poured into the centrifuge tube and a mixture of ethanol and toluene (volume ratio 1:1) was added into the solution. Then centrifuge the solution at the speed of 12,000 r/min for 5 min and pour out the liquid. Add in 20 ml the mixture of ethanol and toluene (volume ratio 1:1), and centrifuge at the speed of 12,000 r/min for 5 min. Finally, the WTe_2_ nanoflowers were added in ethanol for storage.

#### Preparation of WTe_2_ nanoflakes

0.1 mmol W(CO)_6_ was added into a 50-ml round-bottomed flask; then, 10 ml oleyl amine was added into the same flask and mixed with W(CO)_6_. Pump out the air in the flask, introduce N_2_ gas flow at room temperature and stir with magnetons for 3 times. Then 2 ml TOP-Te and 0.5 ml HMDS were injected into the flask. Then the flask was heated to 300 °C and kept for 30 min. Then the WTe_2_ nanoflakes were separated by the similar centrifuge process and stored in the ethanol solution.

#### Preparation of WTe_2_/carbon hybrid catalysts

0.1 mmol W(CO)_6_ was added into a 50-ml round-bottomed flask; then, 10 ml oleyl amine was added into the same flask and mixed with W(CO)_6_. Sonicate the flask for 10 min. Then add 6 mg graphene oxide or CNTs and sonicate for 30 min. Pump out the air in the flask, introduce N_2_ gas flow at room temperature and stir with magnetons for 3 times. Then 2 ml TOP-Te and 0.5 ml HMDS were injected into the flask. Then the flask was heated to 300 °C and kept for 30 min. Then the WTe_2_/carbon hybrid catalysts were separated by the similar centrifuge process and stored in the ethanol solution.

#### Sample characterization

The crystal structures of WTe_2_ nanostructures were characterized by X-ray diffraction (Rigaku Smartlab) with Cu Kα radiation to analyze the phase composition of the as-synthesized samples. Raman scattering spectrum (Horiba LabRAM) was used to characterize the chemical bonds vibration modes of the as-synthesized WTe_2_ nanostructures with the excitation laser of 532 nm to analyze the molecular structures. A Bruker Fourier transform infrared spectrometer (Vertex 70) integrated with a Hyperion 2000 microscope system was used to obtain the spectra, in which the measured range is from 600 to 6000 cm^−1^ to illustrate the adsorbed organic groups. The scanning electron microscope (Hitachi SU8230) has been used to obtain the microstructures of the as-synthesized samples. The crystalline structures and elemental composition distribution were characterized by using the transmission electron microscopy (FEI Tecnai Osiris) and high-angle angular dark-field–scanning transmission electronic microscopy (HAADF–STEM).

### Electrochemical measurements and microreactor fabrication

The electrochemical performance of all samples was tested by the linear sweep voltammetry (LSV) using standard three-electrode systems with the CHI760E electrochemical workstation. High-surface-area carbon fiber papers (CFP) were used as current collectors and catalysts loading substrates, in which the WTe_2_ nanostructures were mixed with Nafion films, ethanol and water, finally drop-cast on the CFP and dried under the infrared light illumination. The Hg/Hg_2_SO_4_ electrode worked as the reference electrode and the electrolyte was the 0.5 M H_2_SO_4_ solution.

Thin exfoliated WTe_2_ flakes and single-layer CVD graphene were transferred on onto the 300 nm SiO_2/_Si substrate to form the heterostructures. The device is fabricated via an Ultraviolet Maskless Lithography machine (TuoTuo Technology (Suzhou) Co., Ltd.), to define electrochemical reaction areas and electrode areas. Thermal evaporation under high vacuum (10^–6^ ~ 10^–7^ torr) was used to deposit the gold electrodes. A second lithography was performed to define photoresist windows, which exposed specific areas for HER after developing. During the HER measurements (scan rate: 5 mV s^−1^, 0.5 mol L^−1^ H_2_SO_4_), we ensure that the gold electrodes are well covered by the photoresist similar with our previous report.

## Results and discussion

**Fig. 1:**
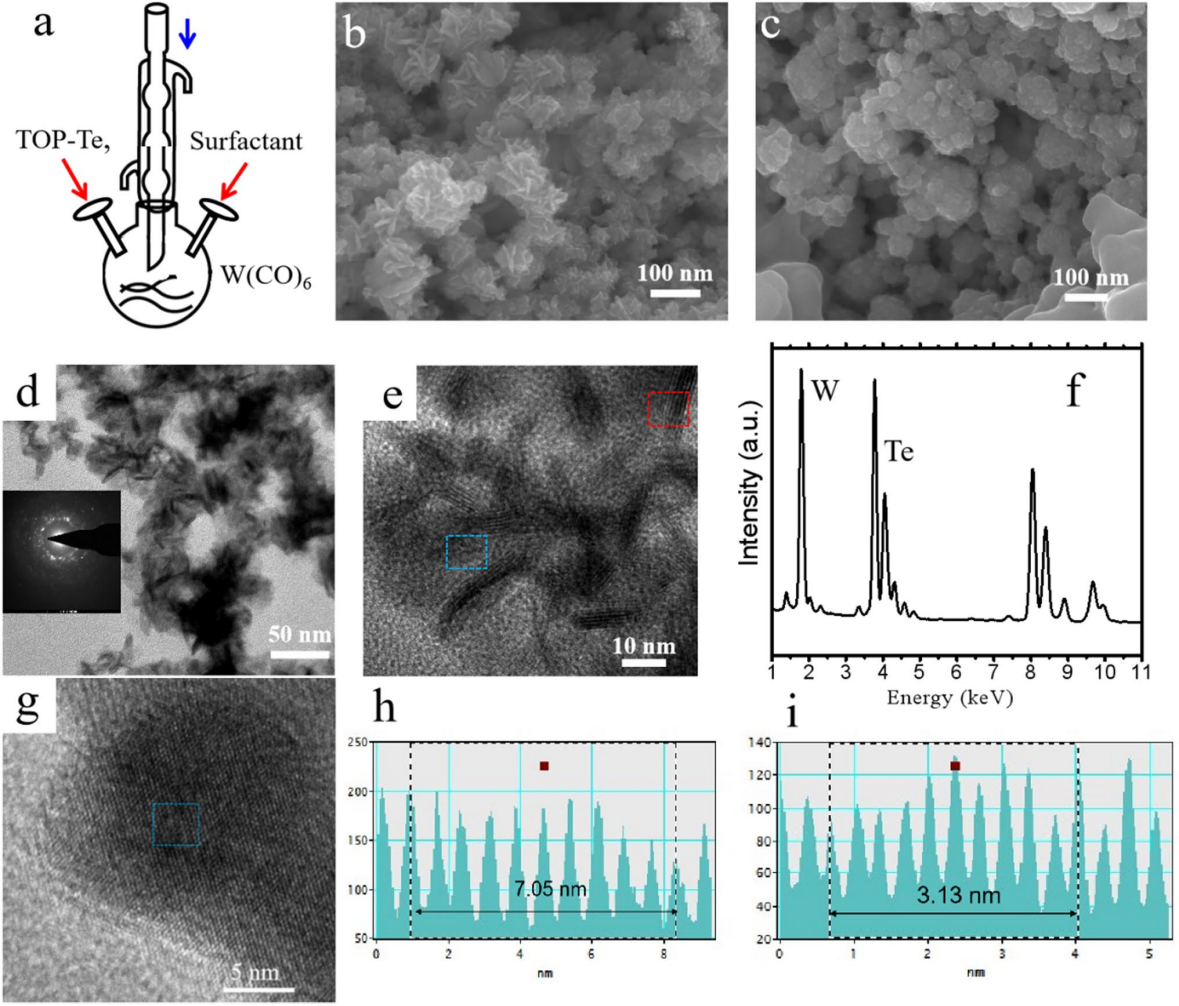
**a** Schematic synthesis of WTe_2_ nanosheets and nanoflowers by tuning the surfactants. **b** Representative SEM images of WTe_2_ nanoflowers formed by the oleic acid and oleyl amine. c Representative SEM images of WTe_2_ nanosheets formed by the oleic acid and HMDS. **d** Representative TEM images of WTe_2_ nanoflowers that transferred on the Cu grid. Inset: selected area electron diffraction of polycrystalline samples. **e** High-resolution TEM image of WTe_2_ nanoflowers with lattice fringes. **f** EDS spectrum of WTe_2_ nanostructures. **g** High-resolution TEM image of WTe_2_ nanoflowers with the lattice fringe. **h**, **i** Measurement of crystal plane distance with the interlayer (0.71 nm) and intralayer (0.31 nm) lattice fringes

Figure 1a shows the schematic growth steps for low-temperature colloidal synthesis of WTe_2_ nanostructures. First, tungsten carbonyl (W(CO)_6_) powders were dispersed into the surfactant solution with/without targeted substrates such as graphene oxide (GO) or carbon nanotubes (CNTs). Next, the prepared TOP-Te solutions were injected into the above solutions by slow rates in few minutes. Then, the growth process was continued for 30 min at 250 ~ 300 °C [37]. Figure 1b shows the scanning electron microscopy (SEM) images of as-synthesized WTe_2_ nanoflowers that used oleic acid and oleyl amine as the surfactant, in which the layers are around 10 nm and are attached together to form the flowers morphology with the sizes of hundreds of nanometers. Figure 1c shows another type of WTe_2_ samples that grown by the injection of oleic acid and hexamethyldisilane (HMDS), stacked as round shape nanosheets. Generally, we could conclude oleyl amine make WTe_2_ tend to grow along the in-plane direction to form the nanoflowers, rather than the role of HMDS making the out-of-plane growth direction. However, more systematic investigations are needed to unravel the growth mechanism, which could be our future work. To explore the microstructure and chemical composition of as-synthesized WTe_2_ samples, the TEM characterization has been carried out. Figure 1d shows the low-resolution TEM image of WTe_2_ nanoflowers, which indicates the WTe_2_ nanoflowers could be separated as ultrathin layers and may be grown with different crystal orientations. The inset image of Fig. 1d shows the selected area electron diffraction (SAED) with random diffraction spots, revealing the dispersed WTe_2_ layers are polycrystalline [27]. Figure 1e shows the high-resolution TEM image of WTe_2_ nanolayers, in which the lattice fringe is clearly shown with the interlayer distance of 0.71 nm (Fig. 1h) and the intralayer distance of 0.31 nm (Fig. 1g and h). Those explanation could be identified with horizontal and vertical crystal growth and are consistent with our previous CVD synthesis reports [27]. The more TEM detailed morphology of as-grown WTe_2_ nanosheets (Fig. 1c) is shown in Fig. S1. Figure 1f shows the energy-dispersive X-ray spectrum of as-synthesized sample, revealing the atomic ratio of W/Te of as-synthesized WTe_2_ nanostructures is around 0.5 ± 0.02, in agreement with previous XPS results [27]. Therefore, both the identification of crystal structures and chemical compositions confirm the T_d_ phase of as-synthesized WTe_2_ samples.

**Fig. 2:**
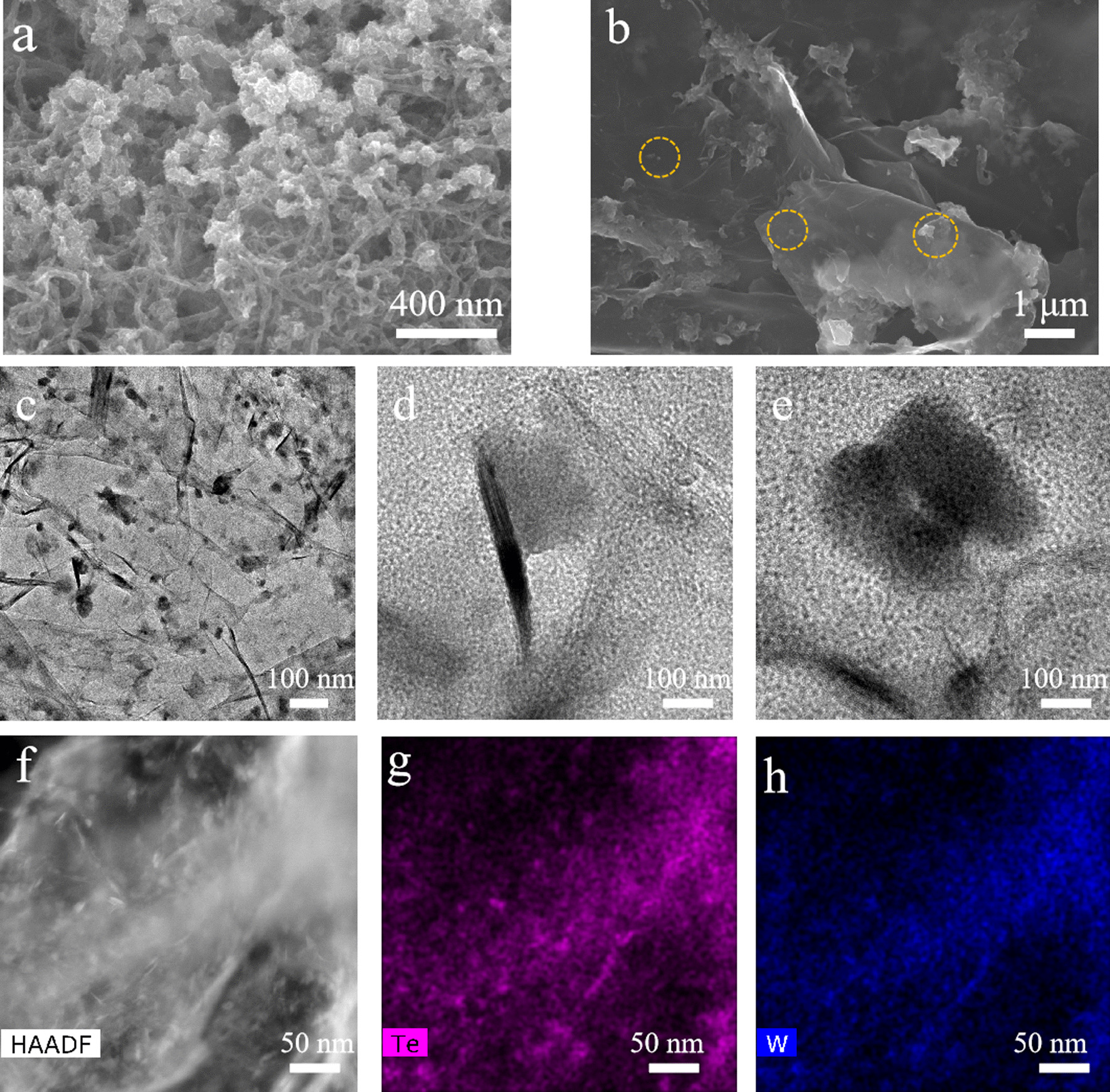
**a** Representative SEM image of WTe_2_ nanosheets that grown on the carbon nanotubes. **b** Representative SEM image of WTe_2_ nanosheets that grown on the reduced graphene oxide layers. **c** Low-resolution TEM images of WTe_2_ nanocrystals grown on the GO. **d**, **e** High-resolution WTe_2_ nanocrystals that vertically and horizontally grown on the GO layers. **f** HAADF–STEM image of the GO–WTe_2_ hybrids. **g**, **h** HAADF–STEM EDX mapping for Te and W, respectively

To further explore if the specific electrochemical interface could improve charge transfer process and promote the overall HER performance, the carbon-based WTe_2_ hybrid structures were synthesized [38, 39]. It is worth mentioning that carbon nanotube and graphene oxide could serve as the novel substrates for anchoring WTe_2_ nanostructures due to the rich chemical groups at the surfaces and edges such as hydroxyl and carboxyl groups [11]. Figure 2a and b shows the SEM images of as-grown CNT–WTe_2_ hybrids and GO–WTe_2_ hybrids. The growth of WTe_2_ nanostructures on the GO layers was found to be more selective than that on the CNTs, in which the numbers of free particles in the CNT–WTe_2_ hybrids are far more than the GO–WTe_2_ hybrids [11]. This growth difference could be ascribed to stronger chemical interactions between the GO–WTe_2_ functional groups and much larger surface areas than that of CNT–WTe_2_ hybrids. More detailed growth results are shown in Fig. S2. High-resolution TEM characterization has been performed to measure the crystal distributions and anchoring states of WTe_2_ nanostructures on the graphene oxide layers [40]. Figure 2c shows the low-resolution TEM image of as-grown WTe_2_–GO nanostructures, in which the large density of WTe_2_ nanoflakes was identified at the wrinkles or edges of graphene oxide layers that are due to the existence of various defects [10]. Figure 2d and e shows the selective areas of as-grown GO–WTe_2_ nanostructures, clearly demonstrating the two different crystal growth directions. The lattice fringes of 0.71 nm and 0.31 nm were identified (002) crystal plane and (020) crystal plane, respectively [27]. The random growth orientation of WTe_2_ nanostructures on graphene oxide layers not only indicates the rich catalytic sites on the surface, but also shows the efficient charge transfer through the vertically grown WTe_2_ layers [26, 31]. All of those morphological characteristics and crystal orientations could boost the HER performance. Figure 2f shows a low-magnification high-angle annular dark-field (HAADF)–scanning transmission electron microscopy (STEM) image of GO–WTe_2_ hybrids, in which a large amount of tiny WTe_2_ nanocrystals were randomly distributed on the graphene oxide layers. Spatially resolved elemental EDX mapping of W and Te in Fig. 2g and h shows that as-synthesized GO–WTe_2_ hybrid catalysts have a large nucleation and anchoring nanocrystals sites on the layers, which could provide a lot of protons adsorption sites and accelerate hydrogen evolution reaction rates.

**Fig. 3:**
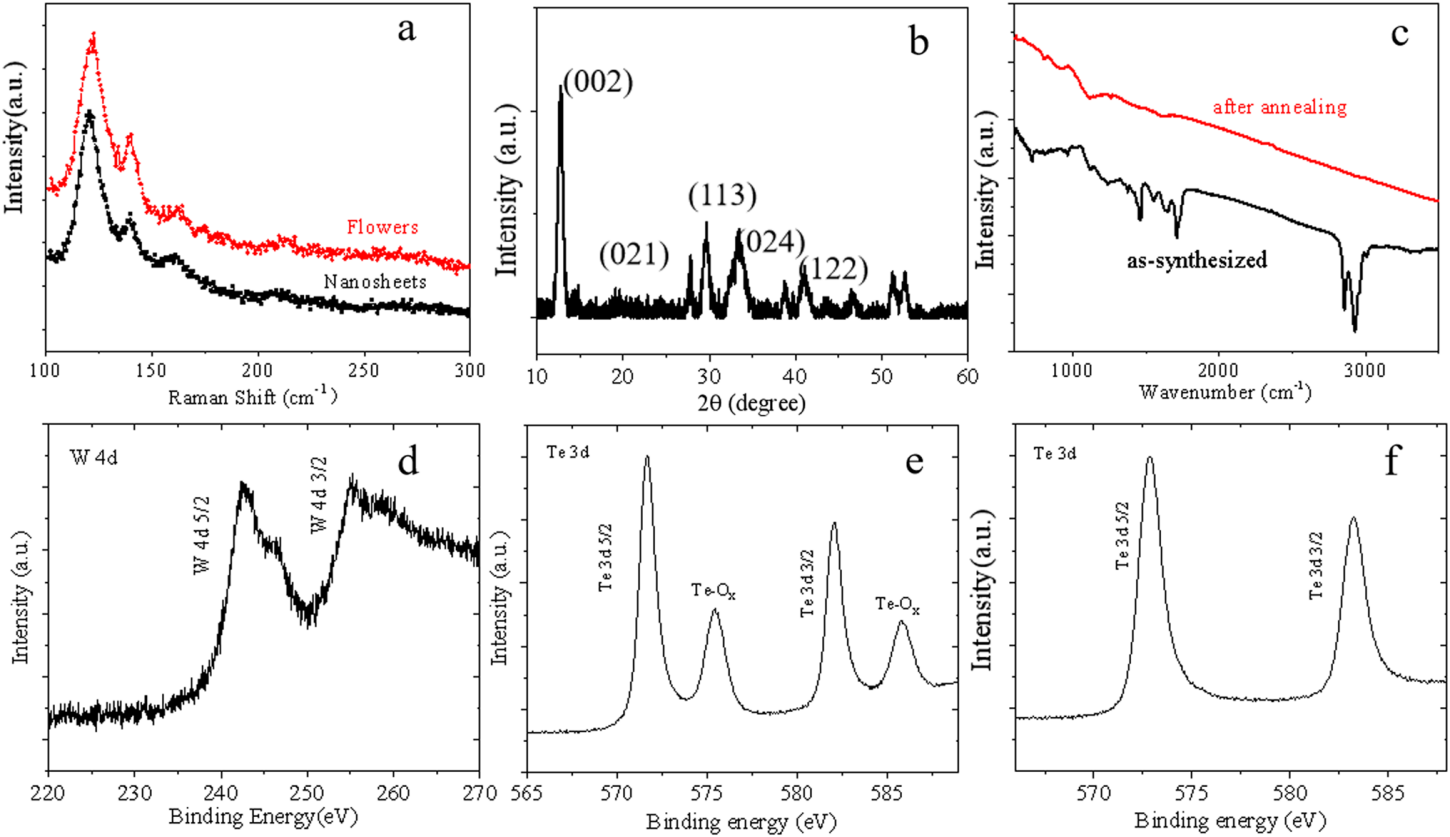
**a** Raman spectrum of WTe_2_ nanosheets. **b** X-ray diffraction patterns of WTe_2_ nanosheets. **c** Infrared spectrum for the as-synthesized and the annealed WTe_2_ nanosheets. **d**, **e**, **f** W 4d (4d_5/2_, 242.3 eV; 4d_3/2_, 254.8 eV) and Te 3d (3d_5/2_, 571.8 eV; 3d_3/2_, 582.2 eV) XPS spectrum of as-synthesized WTe_2_ nanosheets (**d**, **e**) and after electrochemical reaction (**f**)

The crystal phase of WTe_2_ was checked by X-ray diffraction and spectroscopic investigation, which is important to study the structure–performance relationships. Figure 3a shows the Raman spectra of WTe_2_ nanostructures, which could assign the peaks at 120 cm^−1^, 140 cm^−1^ and 160 cm^−1^ that correspond to recent reports [27, 41–43]. The rich Raman modes also indicate the crystal growth orientations are different with the horizontal nanoplates. The GO–WTe_2_ hybrids and WTe_2_ nanosheets are characterized by X-ray diffraction (XRD) in Figs. 3b and S3, and the broad diffraction peaks (Fig. 3b) indicated nanosized WTe_2_ crystal domains with a distorted 1 T structures that results in metallic physical properties. The Infrared spectrum of as-grown hybrids clearly shows the C = O groups (1750 cm^−1^) and CH_3_ groups (2870 cm^−1^ and 2960 cm^−1^), in which the adsorbed chemical groups disappeared after thermal annealing in Fig. 3c [44]. The HER electrochemical properties of as-grown hybrids catalysts could be improved by removing these adsorbed groups that hindered the intrinsic catalytic sites. Figure 3d, e and f shows the XPS spectrum to check the surface composition and oxidation states of as-synthesized WTe_2_ catalysts, where the W 4d_5/2_ and 4d_3/2_ peaks are located at 242.3 eV (2*d*_5/2_) and 254.8 eV (4_*d*3/2_), Te 3*d*_5/2_ and 3*d*_3/2_ peaks are located at the binding energy of 571.8 and 582.2 eV. Figure 3e shows the obvious oxidation satellite peaks, which clearly illustrate the unstable nature for telluride thin layers after the storage under air environment. However, WTe_2_ nanostructures could expose the fresh surface of W and Te atom without oxidation after electrochemical reaction, which confirms by the XPS data (Figs. 3f and S4).

**Fig. 4:**
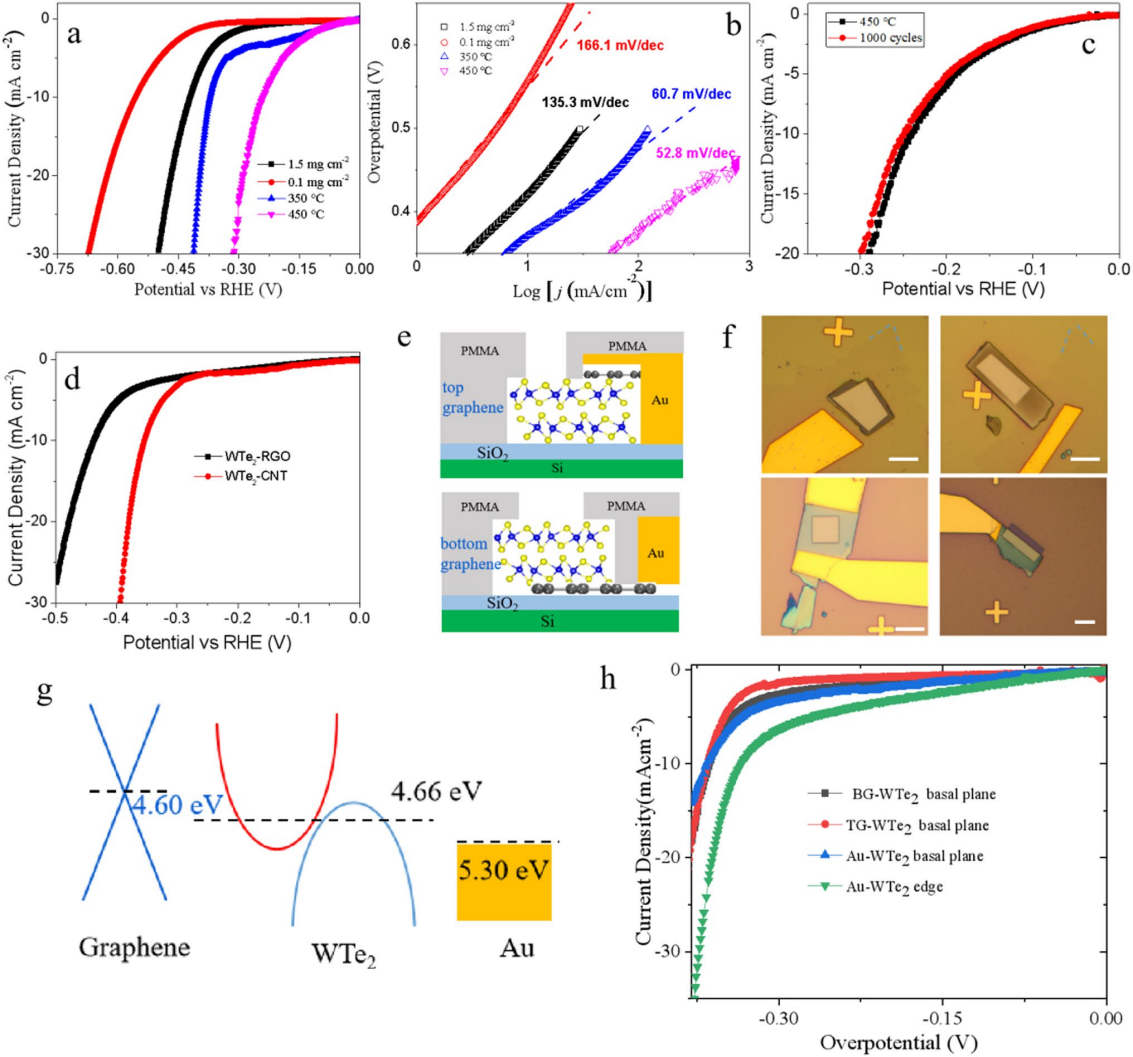
**a** Polarization curves of WTe_2_ flowers that prepared on carbon fiber paper. 0.5 M H_2_SO_4_ solution was used as the electrolyte (scan rate: 5 mV s-1). **b** Corresponding Tafel plot. **c** Cycling measurements of WTe_2_ flowers that annealed at 450 °C (**d**) Comparison of the performance of WTe_2_–GO and WTe_2_–CNT samples. **e** Schematic of electrochemical microreactor for graphene top-contacted WTe_2_ and graphene bottom-contacted WTe_2_ devices. **f** Optical images of electrochemical microreactor for BG–WTe_2_ basal plane device, TG–WTe_2_ basal plane device, Au–WTe_2_ basal plane device and Au–WTe_2_ edge device, respectively. **g** Energy diagram for gold, WTe_2_ and graphene. **h** Polarization curves for BG–WTe_2_ basal plane device, TG–WTe_2_ basal plane device, Au–WTe_2_ basal plane device and Au–WTe_2_ edge device

As reported previously, theoretically 2D WTe_2_ layers could have shown high intrinsic HER catalytic performance at the Te and W edges [26]. Here we firstly explore its intrinsic HER performance systematically, including the contribution of active sites and interfacial charge transfer process [26]. For HER measurements of various WTe_2_ nanostructures, the standard three-electrode setup were used to perform the electrochemical tests. The high-surface-area carbon fiber papers (CFP) were used as current collectors and catalysts loading substrates, in which WTe_2_ nanostructures were mixed with Nafion films, ethanol and water, finally drop-casted on the CFP and dried under the infrared light illumination [45, 46]. The typical cathodic polarization curves of as-synthesized WTe_2_ nanoflowers with different loading amounts and annealing temperatures are shown in Fig. 4a. Mass loading is selectively estimated to be ~ 1.5 mg cm^−2^ and 0.1 mg cm^−2^ (for samples annealed at 350 and 450 °C), respectively, which is for the performance comparison. As the loading mass increases, the over-potentials at the reduction current density of 10 mA cm^−2^ shift with a large reduction value of 140 mV (Fig. 4a). The commercial Pt/C has been be used as the comparison purpose in Fig. S5. To show the intrinsic HER catalytic performance, the thermal annealing process was carried out for as-synthesized WTe_2_ nanoflowers, which makes the catalytic active sites could expose to the electrolyte solution, not covered by the chemical ligands. For comparison, we measured HER of as-synthesized WTe_2_ nanostructures under different annealing temperatures (Fig. 4a). As shown in Fig. 4a, the onset potential and overpotential of higher-annealing-temperature samples are much better than that of lower annealing temperatures samples, showing with lowest overpotential of 240 mV at the current density of 10 mA cm^−2^. This indicates the removal of chemical ligands after thermal annealing process, exposing more active sites, maintaining the crystal structure that could keep metallic conductivity and also showing with better performance than other high-temperature synthesized samples (Fig. S6).

Figure 4b shows the Tafel slope of the higher mass loading samples is 135.3 mV/dec, much lower than the Tafel slope of the lower mass loading samples, which is 166.1 mV/dec. The Tafel slope of higher-annealing-temperature samples is 52.8 mV/dec, much smaller than that of the lower annealing temperatures samples. The low Tafel slopes of higher-annealing-temperature samples suggest relatively fast kinetics for hydrogen evolution after the removing of chemical ligands. For the extensively studied MoS_2_ and WS_2_, their HER activity has been shown to improve dramatically by converting the semiconducting 2H phase to the metallic 1 T phase via Li^+^ intercalation and exfoliation. Atomic strain to obtain distorted 1 T phase, T_d_, from the 1 T phase has shown to further improve HER in the case of WS_2_ [47]. In the case of WTe_2_ nanostructures, the stable crystal structure is already the metallic T_d_ phase. Our previous studies show us the Te and W edge sites, and even the basal planes can contribute to HER activity with similar theoretical performance as MoS_2_ [26]. The Tafel slope determined by the intrinsic property of the materials depends on the rate-limiting pathways of HER. Thus, we attribute the low Tafel slope of 450 °C annealed samples to the semimetallic conductivity of WTe_2,_ which suggests the Volmer–Heyrovsky mechanism according to the low hydrogen coverage by density functional calculation [26]. Thus, the facile electrode kinetics can be ascribed to the better conductivity of WTe_2_, because of the similar hydrogen adsorptions thermodynamics has been clearly shown in the DFT calculations [26]. Stability was investigated by taking continuous cyclic voltammograms in the cathodic potential range [48] (Fig. 4c). The polarization curve after the 1000^th^ cycle almost overlaps with the first cycle curve, which indicates the intrinsic stable performance of WTe_2_ nanostructures (Fig. 4c).

To study the contribution of interfacial charge transfer, the HER performance of CNT–WTe_2_ and GO–WTe_2_ hybrid catalysts was evaluated under the same mass loading conditions (Fig. 4d). The CNT–WTe_2_ hybrid catalysts show the overpotential reduction of 80 mV than the GO–WTe_2_ hybrid catalysts, which is due to better conductivity of CNT–WTe_2_ than GO–WTe_2_. It could be inferred that the lower overall performance of GO–WTe_2_ hybrid catalysts could be attributed to the low mass loading and the agglomeration of WTe_2_. However, this phenomenon seems like different with previous reports. Therefore, the microreactor has been used to explore the interface contribution for electrochemical performance. Figure 4e shows the schematic device structure of WTe_2_–bottom graphene and top graphene–WTe_2_ heterojunction, in which only the catalysts active area was exposed to electrolyte and other areas were covered by photoresists mask. Figure 4f shows the optical images of BG–WTe_2_ basal plane device, TG–WTe_2_ basal plane device, Au–WTe_2_ basal plane device and Au–WTe_2_ edge device, respectively, in which the clear interface was created. (Figure S7 shows the Raman data for single-layer graphene.) The polarization curve for the four different devices is shown in Fig. 4h. In terms of energy diagram in Fig. 4g, graphene was more favorable that gold for serving as the selected substrate because of the better band alignment. However, the Au–WTe_2_ edge device shows remarkable performance than other three kinds of devices, which indicates the charge transfer process is not limited by the any interface. Meantime, the performances of BG–WTe_2_ basal plane device, TG–WTe_2_ basal plane device and Au–WTe_2_ basal plane device are almost the same, suggesting the interlayer charge hopping and interface barrier do not serve as the limiting steps for WTe_2_-based catalysts. Therefore, we can understand the semimetallic WTe_2_ catalysts do not need to the complex interface design compared to other semiconducting layer materials such as MoS_2_.

In conclusion, various T_d_ phase WTe_2_ nanostructures have been synthesized with lateral size control up to the micrometer range by a colloidal synthesis process through different chemical ligands control. The crystal structure and the morphologies of layers were determined by X-ray diffraction methods, Raman spectrum and SEM images. The synthesis process has been applied to the formation of WTe_2_–GO and WTe_2_–CNT hybrid catalysts. The HER catalytic activity of WTe_2_ flowers was characterized with remarkably low overpotential and small Tafel slope compared to previously reported 2H-MoS_2_ after thermal annealing [49]. The facile kinetics of WTe_2_ nanoflowers, reflected in the 52 mV/dec Tafel slope, may be attributed to stability, basal plane and edge active sites and better conductivity. This makes them very appealing for electrochemical devices application by using semimetallic layered 2D materials because of high conductivity and active sites and not harsh interface design. Future directions will include unraveling the role of chemical ligands in the shape control and defect density control of WTe_2_ nanocrystals, normally which requires high temperatures for their 2D growth, thus promoting the electrochemical application of WTe_2_ nanostructures [50–52].

## Supplementary Information

Supplementary file 1 (DOCX 1180 kb)

## Data Availability

Raw data are available upon request from the corresponding author.

## Abbreviations

2D: Two-dimensional
HER: Hydrogen evolution reaction
WTe_2_: Tungsten ditelluride
HMDS: Hexamethyldisilane
SAED: Selected area electron diffraction
SEM: Scanning electron microscopy
TEM: Transmission electron microscopy

## Code availability

Source code is available upon request from the corresponding author.
