# Supplementary material for: Low-temperature synthesis of colloidal few-layer WTe2 nanostructures for electrochemical hydrogen evolution
Source: Discov Nano. 2023 Mar 15;18(1):44. doi: 10.1186/s11671-023-03796-7 (PMC10214922; doi:10.1186/s11671-023-03796-7)
Supplement: Supplementary file 1 — (DOCX 1180 kb) [file 11671_2023_3796_MOESM1_ESM.docx]

**Supporting Information**

**Low-temperature synthesis of colloidal few-layer WTe_2_ nanostructures for electrochemical hydrogen evolution**

Rui Xie^1,2,5,^**^+^**, Wenchen luo^1,^**^+^**, Luwei Zou^1^, Xiulian Fan^1^, Cheng Li^1^, Tiezheng Lv^3^, Jinming Jiang^6,7^, Zhihui Chen^1^, Yu Zhou^1,4,8*^

1. School of Physics and Electronics, Hunan Key Laboratory of Nanophotonics and Devices, Central South University, 932 South Lushan Road, Changsha, Hunan 410083, P. R. China
2. College of Chemistry, Nankai University, Tianjin 300071, P. R. China
3. Research Institute of Automobile Parts Technology, Hunan Institute of Technology, Hengyang, 421002, P. R. China,
4. Powder Metallurgy Research Institute and State Key Laboratory of Powder Metallurgy, Central South University, Changsha 410083, P. R. China
5. Department of Chemistry, Yale University, New Haven, Connecticut 06511, USA
6. Department of Basic Sciences, Air Force Engineering University, Xi’an, 710051,China
7. State Key Laboratory of Solidification Processing, Northwestern Polytechnical University, Carbon/Carbon Composites Research Center, Xi'an, 710072, China
8. State Key Laboratory of Structural Chemistry, Fujian Institute of Research on the Structure of Matter, Chinese Academy of Sciences, Fuzhou, Fujian 350002, PR China

Email: yu.zhou@csu.edu.cn;

+ stands for the equal contribution.

Experimental section:

*Graphene growth and transfer*: The single-layer graphene domain was grown on copper foil (annealed at 1050℃ for 30 min) under the flow of H_2_ (50 sccm) and CH_4_ (0.2 sccm) at 1050 ℃ for 30 min. The transfer process was conducted by spincoating PMMA layer and etching the copper foil with FeCl_3_ soultion.

*WTe_2_ flakes from bulk crystals:* Bulk WTe_2_ crystals were grown by chemical vapor transport (CVT) method, following our previous report*.* WTe_2_ flakes were then obtained by mechanical exfoliation.


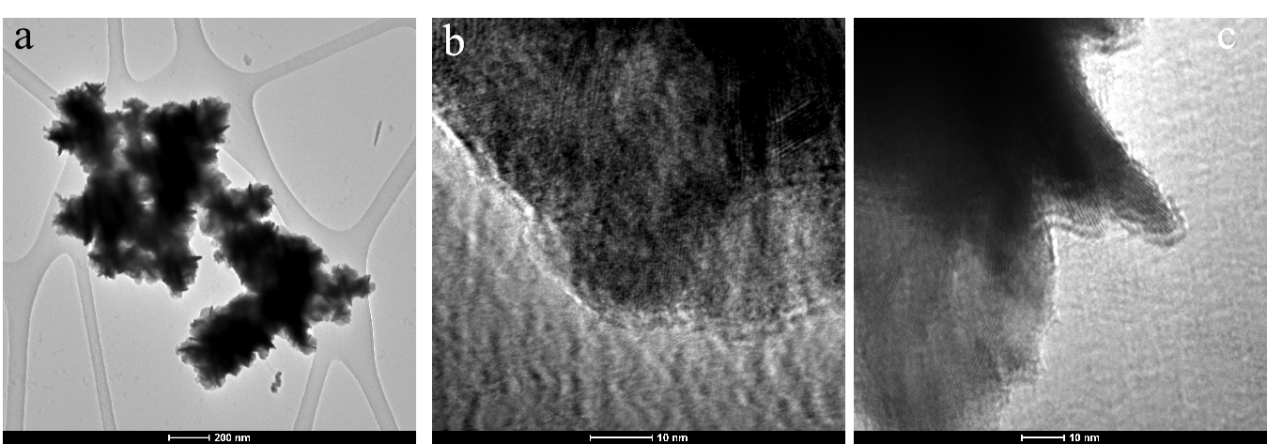


Figure S1. (a) Representative TEM images of WTe_2_ nanosheets that transferred on the Cu grid. (b,c) High resolution TEM image of WTe_2_ nanosheets with lattice fringes


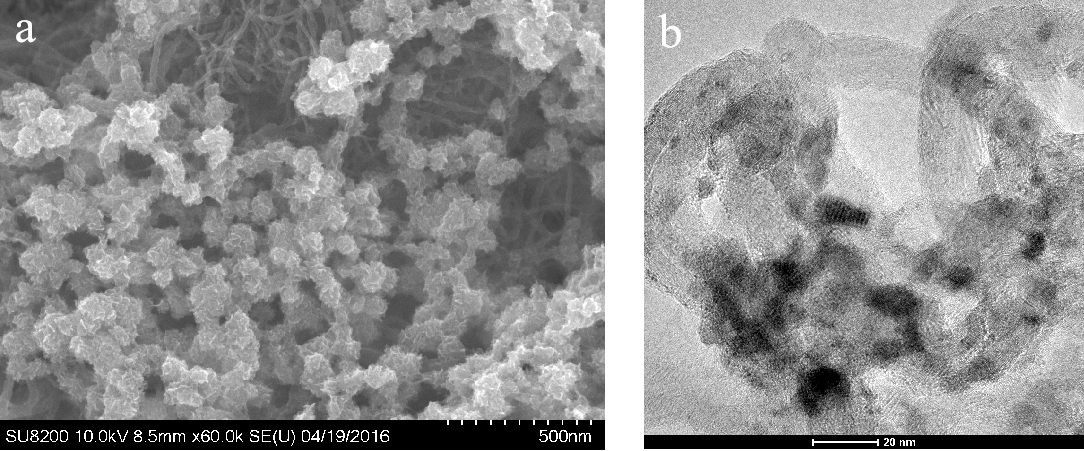


Figure S2. Representative SEM images of WTe_2_ nanoflowers that grown on the carbon nanotubes. (b) High resolution TEM image of WTe_2_ nanoflowers that grown on the carbon nanotubes.


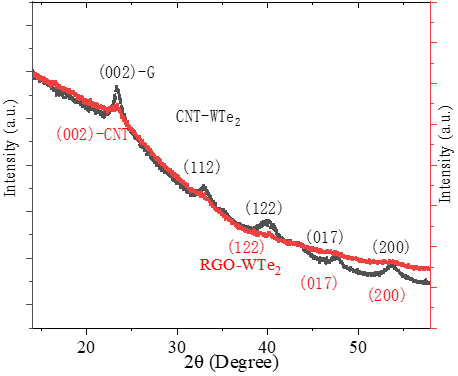


Figure S3. The XRD patterns of carbon-based WTe_2_ nanostructures.


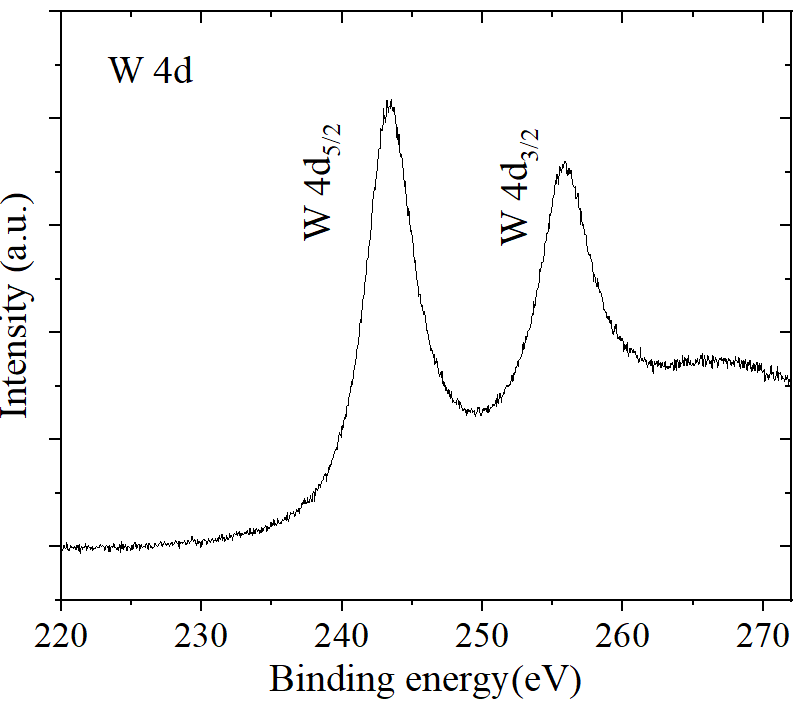


Figure S4. W 4d (4d_5/2_, 242.3 eV; 4d_3/2_, 254.8 eV) XPS spectrum of as-synthesized WTe_2_ nanosheets after electrochemical reaction.


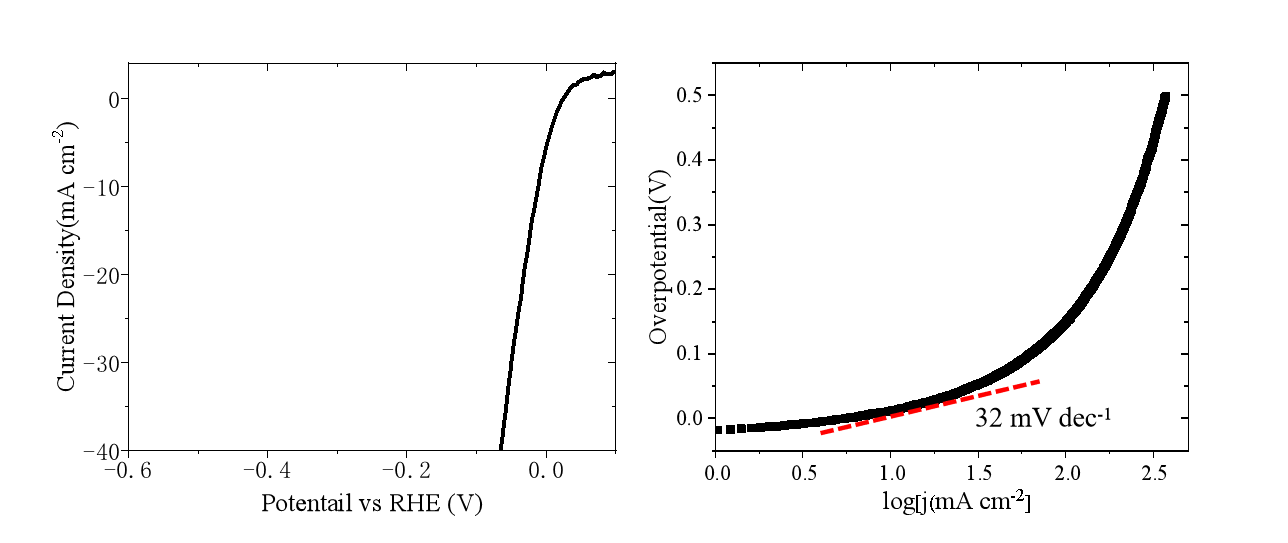


Figure S5. (a) Polarization curves of Pt/C that prepared on carbon fiber paper. 0.5 M H_2_SO_4_ solution was used as the electrolyte (scan rate: 5 mV s^-1^). (b) Corresponding Tafel plot.


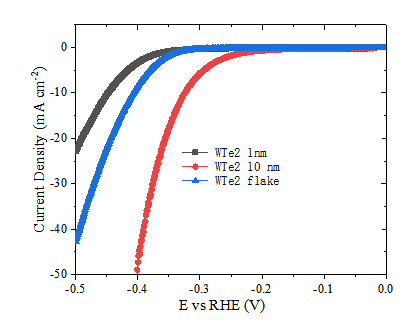


Figure S6. HER performance of WTe_2_ samples that converted from 1 nm and 10 nm W seed layer, single crystals nanoflakes.


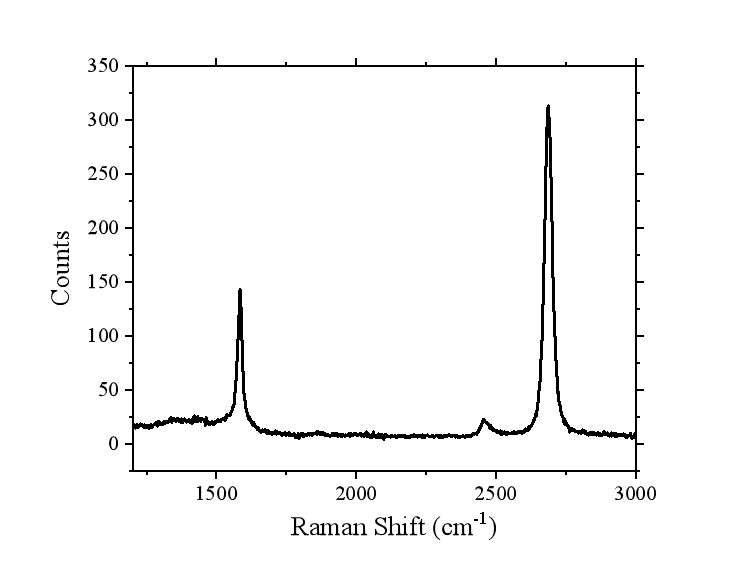


Figure S7. Raman spectrum of single layer graphene transferred on the SiO_2_/Si substrate.
